# Indents

**Published:** 1921-11

**Authors:** 


### INDENTS

**☞Brief Articles of Technical or Scientific Value will receive notice and comment. Contributions invited.**

---

Shall Pulpless Teeth Be Retained in the Mouth? No inflexible rule can be laid down for the retention or extraction of all pulpless teeth, but the decision must be guided by the physical condition of the patient, the condition of the apical and periapical tissues, and the skill of the operator. Although extraction and root-canal treatments may remove infection, the prevention of even small cavities and the maintenance of the gums in a healthy condition must be the aim and object of progressive dentistry. The extraction of a tooth, breaking the arch, produces a condition inviting infection about other teeth due to the establishment of faulty contacts. No artificial device has yet been worked out to replace missing teeth, which is not an irritant to dental or peridental tissue. Physicians and roentgenologists should abandon their present practice of diagnosing oral conditions from radiograms alone and advising the extraction of teeth without consultation with the dentist. The latter should report oral foci of possible systemic infection to the patient and the physician. An effort should be made to eradicate all such foci. Extraction and curettage is the quickest and probably the most certain method of eradicating apical abscesses. A very large percentage of apical radiolucent areas disappear following careful root treatment. Physical symptoms seem to disappear following careful treatment of septic teeth. Before restoring to extraction, attempts should always be made to cure and preserve teeth which are infected. Many physicians have found it much easier to discover a few shadows on dental films than to make a thorough physical examination, and the dentist has found it easier and more profitable to have a tooth extracted and bridge the space than to spend the necessary time in doing a thorough root-canal treatment. It is impossible for either the physician or the roentgenologist to interpret conditions accurately from radiograms alone. But radiograms of all pulpless teeth in the mouths of the dentist's patients should be made at least once each year and the condition be compared with that existing before. Further treatment must be governed by individual requirements.—M. E. Peters, *Cosmos* 1921.

---

MORE FREQUENT CONSULTATION. The first concern of both medical and dental practitioners should be "service to the patient," and the best possible service in many cases means frequent consultation and cordial co-operation covering both pathological and physiological phases of the case.

A prominent internist, who has devoted considerable attention to dental infection and systemic disease, and who might be fairly regarded "a hundred percenter" in his attitude toward dental infection, stated that the more he associated with dentists, and the more he conferred with them in the interpretation of dental radiograms, the less dogmatic he became in his own judgments. The interesting question is, just why should he, as a physician, have been dogmatic at any time in his opinions regarding the teeth and surrounding parts?

We need, in the interests of our patients, more frequent consultations between physicians and dentists, and we also need more frequent consultations between the members of the Dental Profession themselves.—*Oral Health.*

---

Gum Chewing a Bad Habit. I must take issue with the suggestion that gum-chewing is not a bad thing. To my mind gum-chewing is a most pernicious habit and a mental distraction for both young and old. If I mistake not, gum-chewing was suggested by Dr. Kells as something that might take the place of, or that might follow, the improper mastication of food, with a view to correcting the harm done by the bolting of food. But I submit that it would be much better for the dental profession to take the position that the important question is the mastication of food, rather than to suggest that the evils following the lack of proper mastication might be overcome by the artificial operation of gum-chewing.

Gum-chewing is a prevalent habit because nature calls insistently for adequate exercise of the muscles of mastication. If we proprly masticate our food, if we give our masticatory apparatus proper exercise, the bad habit of gum-chewing would not be so prevalent. It is because we bolt our food, it is because of the quick lunch habit (which we have more or less acquired in these days of "rush") that nature is calling for that exercise which we are not supplying. The result is the gum-chewing habit.

I feel that as a profession we should take a position opposed to all of these substitutes for "nature's way." I believe that with correct food, properly prepared and thoroughly masticated, we would not be required to resort to artificial means for the prevention of dental disease, and it is this phase of the question that the dental profession should stress. We are forced to use dentifrices, much as we have to use soap to cleanse the hands and bodies; but we cannot expect these artificial measures to displace the natural cleansing of the teeth by natural mastication functions. Dentifrices, liquids, and chewing gums are poor substitutes for the grinding of foods. Excessive insalivation fatigue the salivary glands and in time works serious injury.—Wallace Seccomb, in *Journal, N. D. A.*, page 620.

---

Importance of Oral Hygiene During Childhood. Study of many skulls of children from 200 to 300 years old of native North Americans preserved in the National Museum showed but a solitary carious tooth. In contrast with this showing the author cites the fact that over 7000 children in West Virginia presented over 16000 unfilled cavities, or over 2 per child. In a large number the lower jaw was undeveloped. The prospect is that in a few generations the civilized white will be edentulous. Underweight children seem to suffer especially from caries, malocclusion and pathological gums. Next to defects of vision, defective teeth was the most common cause for rejection in the late draft. National and State programs for the conservation of the teeth are under way or soon will be in England, New Zealand, New York, Tennessee and West Virginia. There are also similar movements in some cities and in Bridgeport it is estimated that the amount of caries has been reduced 50 per cent, with the prospect that the percentage will reach 70 or 80. But this concerted attempt to secure mouth hygiene has an even greater function for it has been shown in Bridgeport that the incidence of contagious diseases of childhood has been greatly reduced, showing that a dirty mouth favors the breeding of ordinary disease germs. The author mentions the claims of Cotton of Trenton that in the State Insane Asylum many cases of insanity have been benefited by abolishing infected tonsils, tooth roots and similar structures which harbor germs.—H. B. Butler, *American Journal Public Health,* April, 1921.

---

PERIODONTOCLASIA. There are many unsolved problems in periodontia. Light is needed as to the nature of the early pathological changes at the gingival margin: likewise as to the phenomena causing abnormalities of the cemento enamel junction and enamel of the teeth. However, I believe the following conclusions are reasonably established and accepted as such by all periodontists:

1. Periodontoclasia is a curable disease, as old as civilization, and is caused by local mechanical irritants (rough surfaces).

2. It has its beginning in nearly all cases between the ages of eight and eighteen, but is seldom recognized by the dentist before the second or third stage.

3. It is a progressive disease; once established, resolution never takes place until thorough surgical procedure has been employed.

4. It is a disease in which medication of any sort is worse than useless; all that is necessary is to remove the cause, and Nature, the great healer, will effect the cure.—G. R. Lindsay in *Dental Facts.*

---

TESTS OF PINE PRODUCT DISINFECTANTS. The disinfectant action, method of production, and chemical properties of pine-oil and pine-distillate product emulsions are reported in United States Department of Agriculture Bulletin No. 989, by the Bureau of Chemistry and the Insecticide and Fungicide Board, as the result of a bacteriological and chemical study of these products.

The work was undertaken for the purpose of determining the physical, chemical, and disinfectant properties of pine-oil and other pine-distillation products, in order to securc data to assist in the detection of the adulteration of commercial products as well as to check up the statements concerning the deterioration of pine-oil disinfectant and its peculiar behavior against certain pathogenic organisms.

The results reported will be of interest to bacteriologists and chemists who are concerned with testing pine-oil and pine-distillate product emulsions and to hospital authorities, dentists, sanitarians, and others who use these products as disinfectants. The investigators found that these products, while effective against *B. typhosus,* are not effective against *M. aureus* and *B. anthracis,* and should not, therefore, be used for general disinfecting purposes. When using pine-oil emulsions against *B. typhosus* it is safer for practical purposes, according to the report, to employ a solution five times the strength capable of killing the organism in five minutes. Thus, a product showing by the Hygienic Laboratory method a killing power of one-five hundredth should be used in a one-one hundredth, or one per cent dilution. If the product will not give a dilution of such a concentration and remain completely emulsified, it should not be used as a disinfectant.

Copies of Bulletin No. 989, giving data upon which conclusions are based, may be had upon application to the Division of Publications, Department of Agriculture, Washington, D. C.—*U. S. Department of Agriculture.*

---

New Regulations for Narcotic Drugs. The Commissioner of Internal Revenue, under date of October 19th, has issued instructions to narcotic agents and other officials concerned in the enforcement of the Harrison law, amending the instructions issued July 31, 1919, in regard to the application of the law to the treatment of incurable diseases and drug addicts. Regarding the use of narcotics in the treatment of acute disease, the commissioner holds that "without reference to the question of addiction, a physician acting in accordance with proper medical practice may prescribe or dispense narcotics for the relief of acute pain or for any acute condition, such as influenza, pneumonia, renal calculi, broken limbs, etc." This practically gives the physician the right to use narcotic drugs for actual disease conditions, in accordance with the recognized usage of the medical profession.

Regarding the use of narcotics in the treatment of incurable diseases, the commissioner instructs his agents that "a reputable physician directly in charge of bona fide patients suffering from diseases known to be incurable . . . may . . . strictly for legitimate medical purposes, dispense or prescribe drugs for such diseases, provided (1) the patients are *personally attended by the physician*, (2) that he *regulate the dosage*, (3) and *prescribes no quantity greater than that ordinarily recommended by members of his profession* to be sufficient for the proper treatment of the given case." If the patient, through carelessness, secures more narcotics than are necessary, the physician will be held responsible. The prescription must show the date, the full name and address of the patient and describe in indisputable terms the exact nature of the ailment for which issued. It is not lawful, under any circumstances, to place in the hands of an addict, through prescription or otherwise, a sufficient quantity of narcotic drugs to last a week. In incurable, aged, and infirm cases, geographically isolated, the physician may, at his own risk, upon obtaining permission from the narcotic agent in charge of the district, prescribe or dispense a week's supply or more provided it is placed in the custody of a responsible nurse or attendant. Accurate records must be kept of such prescribing and administration.

Regarding the use of narcotics in the treatment of addicts, mere addiction alone is not regarded as incurable disease. The new instructions divide the addicts into two classes: (*a*) those suffering from infirmity or old age, who are confirmed addicts of years' standing and who, in the opinion of a reputable physician in charge of the case, require a minimum amount of narcotics in order to sustain life. Such addicts may be treated in the same manner as those suffering from incurable disease. A responsible physician may prescribe or dispense the minimum amount necessary to meet the absolute needs of the patient. The physician will be held responsible for the results. The physician issuing such a prescription must state on the prescription that the patient is aged and infirm, the age of the patient and the fact that the drug is necessary to sustain life. (*b*) Ordinary addicts must be treated in accordance with the experience of the medical profession, which is that ordinary cases yield to proper treatment, that so-called reductive ambulatory treatment is not effective and that any method of treatment which makes no provision for confinement during withdrawal is a failure in the great majority of cases. The bureau will not, under any circumstances, sanction the treatment of addicts where the drugs are placed in the addicts' possession or where the treatment covers more than thirty days or the patient is not confined in a proper institution. If a physician places narcotic drugs in the possession of an unconfined addict, such action will be regarded as showing lack of good faith. Doubtful cases or those not falling within any of these instructions will, upon request, be investigated and special instructions based upon the recommendations of the inspecting officers will be issued.

---

CANCER PHOBIA. An inevitable accompaniment of directing special attention to any disease is to arouse in a certain number of persons phobias almost as terrifying as the diseases feared. To warn the public that "moles, excrescences, fistulas and warts are the first signs of cancer" is to erect a specter capable of shattering even a normal mentality. To say that "symptoms of indigestion" are "signs of cancer" is so small a part of the whole truth that it is better left unsaid. Carcinoma is in the beginning a local disease. It is definitely known that irritation of tissues favors its production. There has also been accumulated evidence as to the earliest signs by which malignant growths manifest themselves. It is well to give the public such facts as these, to advise early consultation with the family physician, and to point out that we have thus far but one sure method of treatment, and that is complete removal of the dangerous growth. The condition itself is sufficiently serious and needs no elaboration as to its terrifying aspects. Give the public all the facts—but facts only.—*J. A. M. A.*, October 29, 1921.

---

CHEWING OF GUM. Recent statistics show, according to the Internal Revenue Department, that the bill for chewing gum used by the American people has increased last year from 37,000,000 to 44,000,000, making a total of 7,000,000.

I have accordingly asked several authorities about their opinion on the subject, which is here reprinted as follows:

"I doubt if there is any real value in the use of chewing gum, for the reason that the gum is so soft as to require very little chewing force, consequently the friction is practically negligible. I think no one is justified in making a very definite statement regarding sugars until much more scientific work has been done along that line of study. I can see no possible effect of the use of chewing gum in cases of pyorrhea.

"As mandibular movement in gum chewing is only slight, the impact on maxillae is not sufficient to induce increased arterial circulation. If valuable exercise is wanted, it should be obtained by chewing either slippery elm bark or licorice root, preferably the latter, and, again, there would be some cleansing value to the licorice root habit, which is not obtainable from the gum.

"The fermentation from the sugar contained is a negligible factor, because it is soon dissolved and carried away by saliva, and even sweets are harmless in the presence of thorough, faithful and frequent home prophylaxis, practiced with dental floss tapes and suitable brushes.

"Gum chewing would undoubtedly aggravate periodontoclasia from trauma.

"Nature intends that anything chewed should be swallowed. Laws should be passed compelling gum chewers to follow nature's law, and thus rid innocent parties' shoes and clothing of such filth, bearing the still more vicious results of contamination with infected saliva. Jules J. Sarrazin, Arthur B. Black.—M. D. K. Bremner, in *Dental Facts.*

